# Leaky Optoelectrical Fiber for Optogenetic Stimulation and Electrochemical Detection of Dopamine Exocytosis from Human Dopaminergic Neurons

**DOI:** 10.1002/advs.201902011

**Published:** 2019-10-16

**Authors:** Shashank Vasudevan, Janko Kajtez, Ada‐Ioana Bunea, Ana Gonzalez‐Ramos, Tania Ramos‐Moreno, Arto Heiskanen, Merab Kokaia, Niels B. Larsen, Alberto Martínez‐Serrano, Stephan S. Keller, Jenny Emnéus

**Affiliations:** ^1^ Department of Biotechnology and Biomedicine (DTU Bioengineering) Technical University of Denmark Produktionstorvet Building 423, Room 122 2800 Kongens Lyngby Denmark; ^2^ National Center for Nano Fabrication and Characterization (DTU Nanolab) Technical University of Denmark Ørsteds Plads Building 347 2800 Kongens Lyngby Denmark; ^3^ Epilepsy Centre Department of Clinical Sciences Lund University Hospital 221 84 Lund Sweden; ^4^ Lund Stem Cell Center Division of Neurosurgery Department of Clinical Sciences Lund University 221 84 Lund Sweden; ^5^ Department of Health Technology (DTU Health Tech) Technical University of Denmark Produktionstorvet Building 423 2800 Kongens Lyngby Denmark; ^6^ Department of Molecular Biology Universidad Autónoma de Madrid, and Department of Molecular Neuropathology Center of Molecular Biology Severo Ochoa (UAM‐CSIC) Nicolás Cabrera 1 28049 Madrid Spain

**Keywords:** dopamine exocytosis, human neural stem cells, leaky optical fibers, optogenetics, pyrolysis

## Abstract

In Parkinson's disease, the degeneration of dopaminergic neurons in substantia nigra leads to a decrease in the physiological levels of dopamine in striatum. The existing dopaminergic therapies effectively alleviate the symptoms, albeit they do not revert the disease progression and result in significant adverse effects. Transplanting dopaminergic neurons derived from stem cells could restore dopamine levels without additional motor complications. However, the transplanted cells disperse in vivo and it is not possible to stimulate them on demand to modulate dopamine release to prevent dyskinesia. In order to address these issues, this paper presents a multifunctional leaky optoelectrical fiber for potential neuromodulation and as a cell substrate for application in combined optogenetic stem cell therapy. Pyrolytic carbon coated optical fibers are laser ablated to pattern micro‐optical windows to permit light leakage over a large area. The pyrolytic carbon acts as an excellent electrode for the electrochemical detection of dopamine. Human neural stem cells are genetically modified to express the light sensitive opsin channelrhodopsin‐2 and are differentiated into dopaminergic neurons on the leaky optoelectrical fiber. Finally, light leaking from the micro‐optical windows is used to stimulate the dopaminergic neurons resulting in the release of dopamine that is detected in real‐time using chronoamperometry.

## Graphical abstract

On your nerves! Transplanting optogenetically modified human neural stem cell–derived dopaminergic neurons in Parkinson's disease patients could permit continuous modulation of dopamine release from the neurons. This study finds that differentiating neural stem cells on pyrolytic carbon coated leaky optical fiber allows release of dopamine on demand by optical stimulation and its subsequent detection in real‐time using electrochemical techniques.

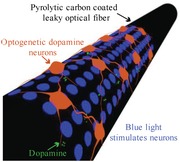

## 1 Introduction

Parkinson's disease (PD) is a progressive neurodegenerative disorder characterized by the degeneration of dopaminergic neurons in the substantia nigra pars compacta (SNpc). This leads to a reduction in the physiological levels of the neurotransmitter dopamine in the target region (caudate‐putamen in the striatum) resulting in the signaling disruption of the nigrostriatal motor pathway, causing both movement impairments and a range of nonmotor symptoms.^1^, ^2^, ^3^ Current medical therapies are aimed at increasing the striatal dopamine levels.^4^ Oral administration of levodopa, a dopamine precursor, has been used for more than four decades and remains the most effective treatment despite its association with an array of complications such as early dose wearing off and dyskinesia.^5^ When medication cannot control motor symptoms, deep brain stimulation (DBS) using electrodes surgically implanted at the targeted region offers therapeutic alternative.^4^ However, the mechanisms through which DBS modulates the underlying brain networks and the effects of local stimulation on brain functioning are still poorly understood.^6^, ^7^, ^8^, ^9^ While these therapies are effective, they are focused on alleviating the symptoms and increasing the quality of life, but do not halt or revert disease progression.^10^

A more neurorestorative approach is cell replacement therapy (CRT), which involves restoration of lost function through the transplantation of dopaminergic neurons in the striatum, to replace dead neurons with healthy ones and to replenish the dopamine levels.^11^, ^12^ Stem cells with their proliferative capacity and ease of in vitro manipulation are believed to be the potential future candidates for CRT.^12^, ^13^ However, CRT alone neither facilitates selective control of the transplanted cells to release dopamine on demand to avoid possible dyskinesia nor allows to monitor their in vivo efficacy.^14^

Optogenetics, a molecular and functional strategy that allows reversible and on‐demand neuronal manipulation by light through the expression of light sensitive ion channels, permits stimulation of genetically isolated neuronal subpopulations. It has been utilized to demonstrate the functional recovery and integration of dopaminergic neurons with the host network in animal models.^15^, ^16^, ^17^ Moreover, optogenetics has been shown to modulate dopamine release in vitro and in brain slices of transplanted animals.^17^, ^18^, ^19^ Due to the electroactive nature of dopamine, it is possible to detect dynamic dopamine release from the neurons using electrochemical techniques.^20^ Electrochemistry has been used to detect fluctuations in dopamine concentrations in vivo and in vitro with high spatial and temporal resolution.^21^ However, the combination of optogenetics and electrochemistry has not yet been used for the detection of in vitro light triggered dopamine release from optogenetic dopaminergic neurons derived from human neural stem cells.

Light delivery in vivo is achieved through an implanted optical fiber.^22^, ^23^ However, standard optical fibers suffer from a small activation volume close to the fiber tip due to the strong attenuation of visible light in the brain tissue.^22^ Clinical recovery in humans would require modulation of a large number of transplanted dopaminergic neurons.^24^ Increasing the input optical power would expand the number of activated neurons, but would also lead to tissue damage due to heat.^25^ Multipoint‐emitting optical fibers have been developed for stimulation of spatially separated neuronal populations.^26^ However, this approach requires a complex coupling strategy at the fiber input. Microfabrication techniques allow monolithic integration of optical and electronic elements but necessitate complex fabrication processes.^27^ Moreover, these devices are used for neural potential recordings and not for the specific detection of released neurotransmitters.

**Figure 1:**
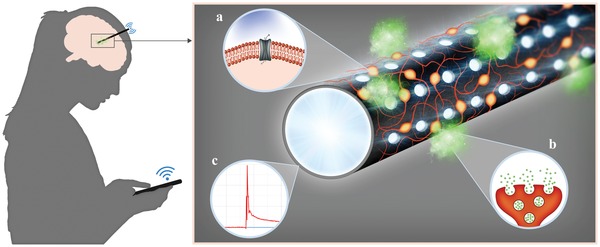
Left: A vision for remote controlled brain bioimplant for personalized and autonomous neurointervention using a multipurpose LOEF. Right: Pyrolytic carbon coated optical fiber (black), serving as a substrate for neural stem cell (orange) differentiation. Micro‐optical windows on the fiber leak blue light that activates a) light‐sensitive ionic channels on the neurons and b) triggers dopamine exocytosis (green). c) The outer conductive pyrolytic carbon allows the use of the LOEF as an electrode for real‐time electrochemical detection of dopamine.

Here, we pursue the vision of a brain bioimplant for autonomous control of dopaminergic neurons derived from optogenetic human neural stem cells for on‐demand light‐induced release of dopamine to restore the striatal dopamine levels (**Figure** **1** (left)). For this purpose, a multifunctional leaky optoelectrical fiber (LOEF) has been developed to simultaneously realize three functions: i) a substrate for the delivery of optogenetic stem cell–derived dopaminergic neurons, ii) an actuator for the optical stimulation of these dopaminergic neurons to release dopamine, and iii) an electrochemical sensor for real‐time detection of the light‐induced dopamine release (Figure 1 (right). We describe the fabrication of a LOEF, a pyrolytic carbon coated optical fiber with an array of light‐emitting micro‐optical windows. The spatial distribution and the intensity of light leaking from the LOEF were characterized and optimized for large volume optogenetic stimulation. The electrochemical properties of the LOEF were characterized using the hexaammineruthenium redox couple and dopamine. The suitability of the pyrolytic carbon coating on the LOEF as a substrate for cell culture was verified by differentiating optogenetic human neural stem cells. Finally, the ability of the LOEF to simultaneously stimulate a large population of optogenetic dopaminergic neurons on its surface by light and subsequently detect the dopamine release was confirmed by chronoamperometry.

## 2 Results and Discussions

### 2.1 Fabrication of Leaky Optoelectrical Fiber

**Figure 2:**
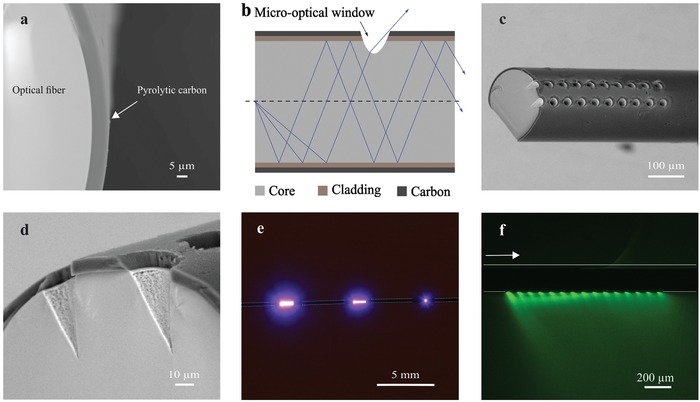
a) SEM showing the cross section of an optical fiber after pyrolysis (8 µm thick pyrolytic carbon layer (dark gray) surrounding the cladding and core (light gray) of the fiber). b) Schematic view of the functional principle of a leaky optoelectrical fiber (LOEF). c) SEM of an LOEF with a 2 × 10 array of laser ablated micro‐optical windows. d) Close‐up of LOEF showing the side‐walls of micro‐optical windows. e) Photo showing different light leak intensity from a pattern of a single, 1 × 10 and 2 × 10 micro‐optical windows (from right to left). f) Image of fluorescent nanobeads in gelatin surrounding the LOEF showing the spatial distribution of light leak. The arrow indicates the direction of light coupling.

A commercially available fused silica‐based optical fiber was pyrolyzed at 900 °C in an inert nitrogen atmosphere. The temperature was ramped slowly at 2 °C min^−1^ to avoid stress build up in the carbon layer. During pyrolysis, the 15 µm thick polyimide buffer layer on the optical fiber decomposes resulting in an ≈8 µm thick pyrolytic carbon layer surrounding the cladding (**Figure** **2**a). The resulting electrically conductive pyrolytic carbon coating has previously been shown to be an excellent electrode material for electrochemical detection of dopamine.^28^

To assess the influence of the pyrolysis process on the optical functionality of the fiber, the optical power transmission of the fiber before and after pyrolysis was analyzed using the cutback method. Light from a blue laser diode (460 nm), the wavelength to be used to optically stimulate optogenetic dopaminergic neurons (excitation peak of channelrhodopsin‐2 (ChR‐2) is 473 nm), was coupled into fiber segments of different lengths (*n* = 3 for each length). There was no change in the transmitted power after pyrolysis. Visual inspection of light coupled into the pyrolyzed fiber indicated no light leakage through the carbon cladding, confirming the absence of any discontinuities in the carbon layer (Figure S1a, Supporting Information). Thus, an optical fiber with both optical and electrical properties is obtained, i.e., an OEF.

In order to convert the OEF into an LOEF, a high‐power picosecond laser was used to ablate micro‐optical windows through the pyrolytic carbon coating, the cladding and into the fiber core. This leads to a local decrease of both incidence and critical angle for the fiber, resulting in light leaking out of the fiber core (Figure 2b). The depth of these micro‐optical windows was optimized by varying the power and frequency of laser pulses during ablation to allow sufficient light to leak out while maintaining the mechanical integrity of the optical fiber. It should be noted that performing the laser ablation before pyrolysis leads to excess thermal stress in the polyimide layer. This stress results in the formation of cracks in the pyrolytic carbon layer post pyrolysis (Figure S1b, Supporting Information). Similar cracks can be observed if the fiber is improperly handled before pyrolysis, e.g., excessive bending of the fiber during cutting. Pyrolyzing the optical fiber before laser ablation eliminates this problem since the heat generated during the laser ablation is dissipated by the carbon layer. Figure 2c shows the scanning electron microscopy (SEM) of the LOEF with an array of 20 micro‐optical windows (two rows of 10 holes each) and Figure 2d shows a close up of the micro‐optical windows. Figure 2e shows the increase in light leak intensity from different patterns of micro‐optical windows (a single, a pattern of 10 and 20 micro‐optical windows). The LOEF was immersed in a dispersion of fluorescent nanobeads and gelatin with blue light (460 nm) coupled into the LOEF. Light leaking from the micro‐optical windows excites the fluorescent nanobeads revealing the spatial distribution of light (Figure 2f). The intensity of light leaking through the 20 micro‐optical windows was measured to be 5.5 mW mm^−2^ when the laser was pulsed at 2 ms (period = 10 ms). This light intensity is sufficient for the optogenetic stimulation as the reported threshold for the activation of ChR‐2 is 1 mW mm^−2^.^22^, ^29^

### 2.2 Electrochemical Characterization of the LOEFs

#### 2.2.1 Response to Hexaammineruthenium(II) and Dopamine

**Figure 3:**
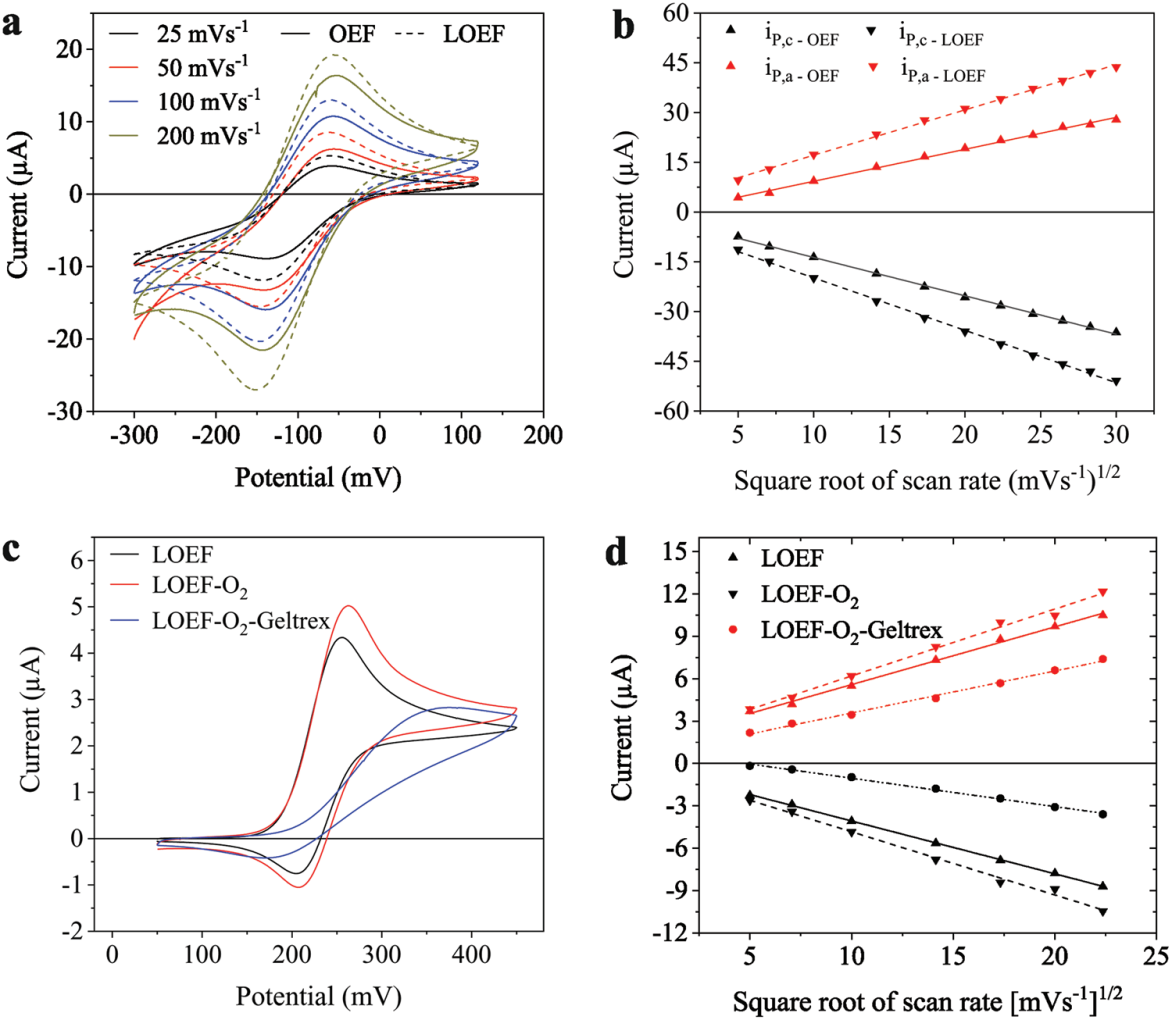
a) Characteristic cyclic voltammograms of 1 × 10^−3^ m [Ru(NH_3_)_6_]^2+^ acquired at different scan rates before (OEF) and after (LOEF) laser ablation. b) *i*_P,a_ and *i*_P,c_ versus the square root of scan rate for the same OEF and LOEF. c) Characteristic cyclic voltammograms of 250 × 10^−6^ m dopamine hydrochloride acquired on an LOEF, showing the influence of O_2_ plasma treatment and Geltrex coating on the electrochemical response (scan rate 50 mV s^−1^). d) *i*_P,a_ (red) and *i*_P,c_ (black) versus the square root of scan rate for the same LOEF before and after O_2_ plasma, and after Geltrex coating.

The OEFs and LOEFs were first characterized as working electrodes in a three‐electrode setup using the outer sphere redox system hexaammineruthenium(II) ([Ru(NH_3_)_6_]^2+^).^30^ A platinum wire was used as the counter electrode and Ag|AgCl (saturated KCl) as the reference electrode. Cyclic voltammograms (CVs) were acquired on the OEFs and LOEFs at different scan rates to assess the influence of the micro‐optical windows on the carbon surface on its electrochemical behavior (**Figure** **3**a; Figure S2a,b, Supporting Information). An increase in the anodic (*i*_P,a_ from 5.5 ± 1.8 to 11.5 ± 1.3 µA) and cathodic (*i*_P,c_ from 11.8 ± 1 to 13.3 ± 1 µA) peak current could be observed for the LOEFs after laser ablation (scan rate ν = 50 mV s^−1^, *n* = 3). A plausible explanation for that could be the marginal increase in the surface area of the carbon due to the exposed vertical sidewalls after laser ablation (Figure 2d) (for a single micro‐optical window, the surface area decrease by laser ablation: 710 ± 38 µm^2^ vs surface area increase due to side walls: 754 ± 20 µm^2^). This increase in currents was accompanied by a marginal decrease in the peak potential separation Δ*E*_p_ (−86.7 ± 3 mV before vs −81.3 ± 3 mV after laser ablation). Previously reported Δ*E*_p_ for photoresist‐derived carbon films under similar pyrolysis conditions as used here was larger than 100 mV.^31^ Both OEFs and LOEFs show a linear relationship with the square root of the scan rate up to 900 mV s^−1^ indicating that the conductivity of the pyrolytic carbon does not limit electrochemical processes at higher scan rates (Figure 3b). Moreover, oxygen plasma treatment has been reported to increase the electron transport resistance of pyrolytic carbon,^32^ however on the LOEFs, we did not observe any significant widening of Δ*E*_p_ with oxygen plasma treatment (before plasma: 81.3 ± 3 vs 80.3 ± 3 mV after plasma at 50 mV s^−1^). The LOEFs demonstrate quasi‐reversible reaction kinetics indicated by the widening of Δ*E*_p_ with scan rate from 80 mV at 25 mV s^−1^ to 117 mV at 900 mV s^−1^ (Figure S2c,d, Supporting Information). Furthermore, these initial results demonstrate excellent electrochemical properties of the LOEF and that laser ablation of the OEF does not deteriorate its electrochemical behavior.

The ability of the LOEFs to oxidize dopamine was investigated by acquiring CVs in dopamine hydrochloride solution. As shown in our previous work, oxygen plasma treatment of carbon surfaces increases the density of oxygen functionalities, which improves the coating of a cell adhesion factor, e.g., Geltrex, required for effective cell adhesion and increases sensitivity toward dopamine oxidation due to the increased density of oxygen functionalities.^28^, ^32^ We characterized the dopamine electrochemistry of the LOEFs before and after oxygen plasma treatment. Acquired CVs on LOEFs before and after O_2_ plasma treatment (Figure 3c; Figure S3a,b, Supporting Information) indicate an increase in *i*_P,a_ from 4.4 ± 0.2 to 4.9 ± 0.3 µA (250 × 10^−6^ m dopamine; ν = 50 mV s^−1^, *n* = 3). The Δ*E*_p_ was slight widened after oxygen plasma (Δ*E*_p_ = 49.6 ± 1.2 mV before vs Δ*E*_p_ = 57.6 ± 3.1 mV after O_2_ plasma). The electrochemical characterization presented above indicates that neither laser ablation of the OEFs nor O_2_ plasma treatment of LOEFs deteriorates the electrochemical performance and that the pyrolytic carbon on the LOEFs is suitable for dopamine detection.

To conduct cell‐based experiments, the LOEFs need to be coated with cell adhesion factors, such as the basement membrane extract Geltrex, to promote cell adhesion. Upon coating oxygen plasma treated LOEFs with Geltrex (Figure 3c; Figure S3c,d, Supporting Information), the electrochemical sensitivity clearly decreased (3.0 ± 0.2 µA, accompanied by a widening of Δ*E*_p_ to 153 ± 9.7 mV). Previous work has shown that extracellular matrix proteins may either increase or decrease sensitivity to dopamine detection. While laminin increased sensitivity to dopamine detection,^33^ collagen decreased the sensitivity in a similar way as we observed for Geltrex.^34^ The decrease in sensitivity may partly be due to the mass transfer barrier formed by a protein coating. Moreover, the free surface charge generated by a protein coating may further explain the observed differences in the electrochemical behavior, showing enhancement of electrochemical sensitivity with laminin. Overall, despite the decrease in sensitivity to dopamine detection caused by the Geltrex coating, the results show that Geltrex coated LOEFs provide sufficient sensitivity for dopamine detection.

#### 2.2.2 Detection of Dopamine Exocytosis upon Potassium‐Induced Depolarization

**Figure 4:**
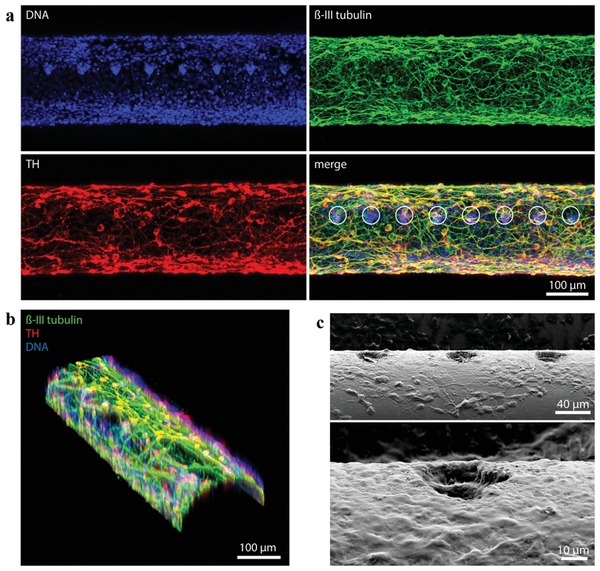
Differentiation of hNSCs into dopaminergic neurons on an LOEF: a) Fluorescence maximum intensity projection of 17 optical sections spanning 100 µm of LOEF. Immunocytochemistry shows an even coverage of the LOEF by the differentiated neurons: ß‐III tubulin (green); TH (red); nuclei (blue); merge (green, red, blue). White circles mark micro‐optical windows. b) 3D reconstruction of the fluorescence images showing cell coverage of the curved surface of LOEF. c) SEM images indicating cell growth on and into the micro‐optical windows of the fiber.

**Figure 5:**
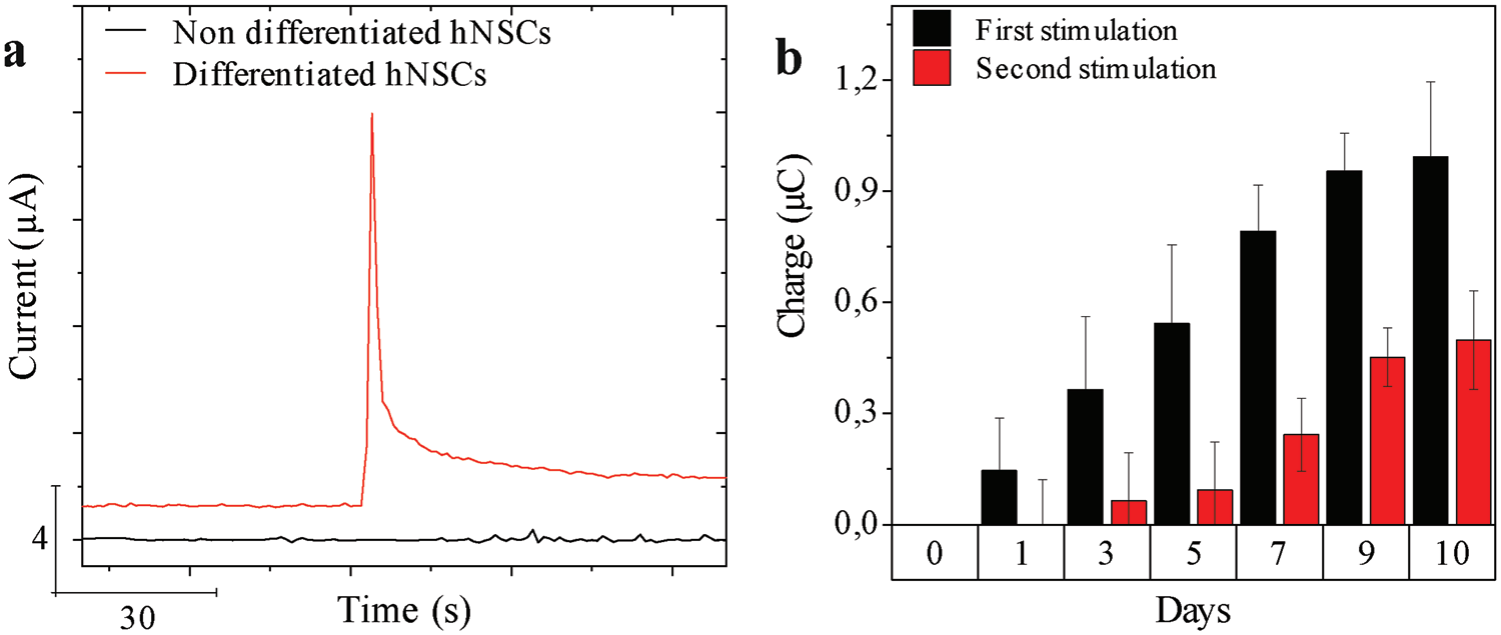
a) Chronoamperometry of dopamine oxidation upon elevation of the final K^+^ concentration to 150 × 10^−3^ m recorded for nondifferentiated (black) and differentiated (red) hNSCs on OEFs. b) Total charge related to dopamine oxidation based on integration of current peaks recorded for differentiating hNSCs during 10 day differentiation period.

We have previously used photoresist‐derived pyrolytic carbon as an efficient substrate for the differentiation of human neural stem cells.^28^ Hence, the ability of the LOEFs to function as a substrate for stem cell differentiation into dopaminergic neurons and an electrode for the detection of dopamine exocytosis was investigated by differentiating human neural stem cells of ventral mesencephalic origin (hNSCs) on the LOEFs, as previously described.^28^ As seen in **Figure** **4**, the LOEF is uniformly covered with a dense neuronal network, as indicated by a high number of β‐III tubulin positive cells (green). More than half (52 ± 7% (*n* = 3)) of the neurons also express tyrosine hydroxylase (TH, red), the rate limiting enzyme involved in dopamine synthesis, indicating efficient differentiation into dopaminergic neurons on the entire surface with a density of 540 ± 73 cells mm^−2^. The neurons grow both on the surface of the LOEFs and into the micro‐optical windows as seen in the SEM images (Figure 4c). The cell culture medium was replaced with a baseline buffer solution containing 5 × 10^−3^ m KCl, followed by the injection of a stimulation buffer to elevate the final KCl concentration to 150 × 10^−3^ m. **Figure** **5**a shows the amperometric current response upon K^+^‐induced depolarization of a population of differentiated hNSCs on the LOEFs. It has previously been shown that the hNSCs used in this study do not differentiate into neurons that produce any other electroactive neurotransmitters besides dopamine.^12^, ^35^ The oxidation of the released dopamine at the surface of the pyrolytic carbon thus results in an increase in the Faradaic current that manifests itself as a peak in amperometry. The current decays to the baseline when dopamine is depleted at the electrode. No current response was obtained when the same experiment was performed using undifferentiated hNSCs due to the absence of dopaminergic neurons. In addition, we monitored the progress of hNSC differentiation and their ability to release dopamine over time by performing chronoamperometry during the differentiation process. Figure 5b shows the determined charges for the first and second exocytosis event within an interval of 15 min (black and red columns, respectively). There was no signal from undifferentiated cells 24 h after seeding (differentiation day 0). However, traces of dopamine could be detected already on day 1 of differentiation, indicating dopamine exocytosis from hNSCs at an early stage of differentiation. As the differentiation progressed over time up to day 10, a continuous increase in signal was observed that can be attributed to the increasing number of dopaminergic neurons. The ratio between the measured charge for the first and the second exocytosis events increased as the differentiation progressed indicating maturation of the differentiated hNSCs. The performed amperometric detection of dopamine exocytosis proves the suitability of the pyrolytic carbon coating as a substrate for stem cell differentiation and as an electrode for electrochemical detection of dopamine exocytosis by potassium‐induced depolarization.

### 2.3 Detection of Dopamine Exocytosis upon Light‐Induced Depolarization

Finally, in order to validate the ability of the LOEFs to optically stimulate the dopaminergic neurons and detect dopamine release, the hNSCs were genetically modified to express the light sensitive opsin ChR‐2 using the human synapsin 1 promoter rendering the transfected hNSC‐derived neurons sensitive to blue light. These hNSC‐ChR‐2 cells were then seeded on the surface of the LOEF and differentiated as described in the previous section.

**Figure 6:**
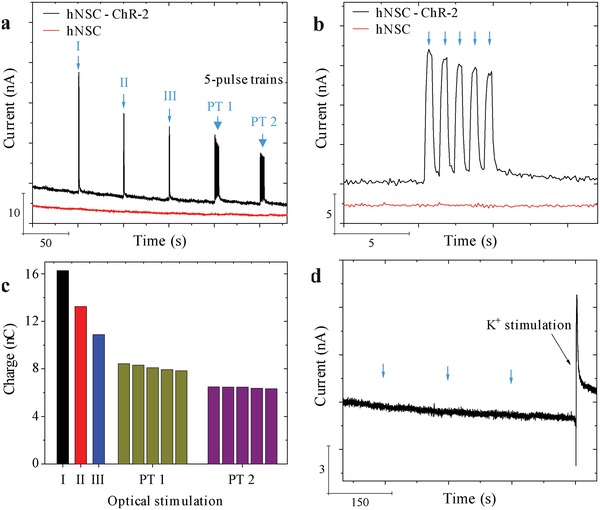
Chronoamperometry of dopamine oxidation from hNSC‐ChR‐2‐derived dopaminergic neurons after 10 days of differentiation: a) Light‐induced depolarization recorded for differentiated hNSC‐ChR‐2 cells (black) and hNSCs (red) on LOEF. From left, three single pulses and two five‐pulse trains (details of pulse width in the text). b) Zoom‐in of a five‐pulse train shown in (a). c) Charge for dopamine oxidation integrated from every peak in (a). d) The effect of light pulses on hNSC‐ChR‐2 cells on an OEF (pulse time indicated by blue arrows) and current peak upon K^+^‐induced depolarization of the same cells.

We modified a 6‐well cell culture plate to allow simultaneous culturing of cells on the LOEF, optical coupling of a laser for optical stimulation and electrical connections to the LOEF for electrochemical recording (Figure S4, Supporting Information). For the light stimulation and electrochemical detection of dopamine, the cell culture medium was replaced with a baseline buffer solution containing 5 × 10^−3^ m KCl. Blue light (460 nm) from a laser diode was coupled into the LOEF carrying differentiated hNSC‐ChR‐2 cells as shown in Figure S4 in the Supporting Information. The stimulation pattern consisted of three single light pulses at an interval of 50 s between pulses followed by three five‐pulse trains with an interval of 50 s between each pulse train. Each pulse had a width of 500 ms and a period of 1 s. Light leaking from the LOEF (white box, Figure S4 inset, Supporting Information) interacts with ChR‐2 eventually triggering the release of dopamine directly at the carbon surface which is immediately detected using amperometry (**Figure** **6**a). Every light stimulation leads to a transient current peak that decays back to the baseline, indicating that light‐induced depolarization triggers dopamine exocytosis. Moreover, the five‐pulse trains of light show the possibility to continuously stimulate the cells within small time intervals and detect the dopamine release from every stimulation event (Figure 6b). This is beneficial for Parkinson's therapy as continuous delivery of dopamine has been shown to reduce levodopa‐induced dyskinesia.^14^ The amplitude of the current decreases with multiple stimulations possibly due to depletion of dopamine in the neurons or ChR‐2 desensitization. This is also visible from the charge measured for every single dopamine oxidation peak (Figure 6c). Simultaneously, nonoptogenetic hNSCs were used as a control. Since these cells are not light sensitive, no change in the baseline current was observed when illuminated by light.

In our setup, the hNSC‐ChR‐2 cells are present both on and around the LOEFs. Hence, it is possible that the dopaminergic neurons close to the tip of the LOEFs could also be stimulated by light leading to dopamine exocytosis (red box, Figure S4 inset, Supporting Information). We have however observed in the past that dopamine is oxidized only when it is released in very close proximity to the electrode surface. It has been shown that a release event as far as 300 nm from the electrode surface does not elicit any changes in the recorded current.^36^ In order to investigate the influence of light leaking from the tip of the LOEFs on the obtained current signal (Figure S4, Supporting Information), we differentiated hNSC‐ChR‐2 cells on OEFs, pyrolyzed optical fibers without laser ablated micro‐optical windows. Light was coupled into the OEFs the same way as in the case of LOEFs and three single optical stimulations, each 50 s apart, were performed. While the light from the tip of the OEF could have stimulated dopaminergic neurons close to the tip, there was no change in the baseline current (Figure 6d). To rule out cell death as the reason for the absence of current peaks, we concluded the experiment by potassium‐induced depolarization of the differentiated hNSC‐ChR‐2 cells.^28^ A current peak was observed that decayed to the baseline, indicating the presence of dopaminergic neurons able to release dopamine. This and the internal controls prove that the signal detected upon light‐induced depolarization was generated by oxidation of the dopamine released from differentiated hNSC‐ChR‐2 cells adhering on the LOEF.

These results provide the first proof of concept for a multifunctional LOEF as a substrate for stem cell differentiation, an actuator for optical stimulation, and a sensor for real‐time electrochemical detection of dopamine exocytosis.

## 3 Conclusion

We have developed and characterized an LOEF for potential use as a brain bioimplant in cell replacement therapy. A commercial optical fiber with a conformal polymer buffer layer was used as the basis to fabricate a pyrolytic carbon coated optical fiber by pyrolysis of the polymer buffer layer. Micro‐optical windows were reproducibly patterned on the surface of the pyrolyzed fiber using laser ablation. Light propagating in the fiber leaks out of these micro‐optical windows due to local change of the critical angle creating a leaky optical fiber. The intensity of light leaking from an array of micro‐optical windows is more than the required threshold intensity for the stimulation of channelrhodopsin‐2. Moreover, the laser ablation does not deteriorate the electrochemical properties of the pyrolyzed fiber thereby turning the pyrolyzed fiber into a leaky optoelectrical fiber. The pyrolytic carbon derived from polyimide consistently displayed excellent electrochemical properties compared to photoresist‐film‐derived pyrolytic carbon demonstrating their reproducibility for use as electrodes. Differentiation of hNSCs on these LOEFs indicates a uniform and dense coverage of the entire surface with differentiated neurons. By chemically depolarizing the neurons using a high concentration of KCl, we were able to detect dopamine exocytosis in real‐time using LOEF as the electrode. This proves the suitability of the LOEFs for stem cell differentiation and an electrode to detect dopamine exocytosis. Furthermore, we genetically modified hNSCs to make them sensitive to blue light to enable selective stimulation of dopaminergic neurons. These optogenetic hNSCs were differentiated on the LOEFs. Optical stimulation of neurons as a result of light leaking from the micro‐optical windows leads to dopamine exocytosis that was detected in real‐time using chronoamperometry. The LOEFs described here thus provide the first proof of concept of a multifunctional device for the differentiation of dopaminergic neurons derived from optogenetic human neural stem cells for on‐demand light‐induced release of dopamine to restore the striatal dopamine levels in Parkinson's disease.

## 4 Experimental Section

*Pyrolysis*, *Laser Ablation, and Optical Characterization of Pyrolyzed Optical Fibers*: Optical fibers with a 200 µm diameter fused silica core, 10 µm thick cladding, and 15 µm polyimide buffer were purchased from Edmund Optics Ltd. (#57‐062). The polymer buffer layer protected the cladding against abrasion. The fiber was pyrolyzed at 900 °C in a furnace (ATV Technologie GmbH, Germany) in an inert nitrogen atmosphere as described previously.^37^

A laser micromachining tool (microSTRUCT vario from 3D‐Micromac AG, 355 nm with a spot size of 15 µm) was used for the microablation of the carbon and the fiber core. The power of the laser was 2 W and the frequency of the pulses was kept at 200 kHz to increase heat dissipation between pulses and to avoid excessive melting of the core material. Optical power measurements were performed using a PM130D power meter (Thorlabs Inc.).

*Electrochemical Characterization of Pyrolyzed Optical Fibers*: All reagents were purchased from Sigma‐Aldrich/ThermoFischer. The electrochemical measurements were performed using a CHI 1010 potentiostat (CH Instruments, Austin TX) with a three‐electrode setup. The OEF/ LOEF were employed as the working electrode, a 500 µm diameter platinum electrode (Advent Research materials Ltd., Oxford, England) as a counter electrode, and a DRIREF‐L Ag|AgCL (saturated KCl) (WPI, Sarasoata, FL) as the reference electrode. The OEFs and LOEFs were characterized by acquiring CVs at varying scan rates in 1 × 10^−3^ m hexaammineruthenium(II) chloride and dopamine hydrochloride (concentrations ranging from 5 × 10^−6^ to 500 × 10^−6^ m) solutions prepared in phosphate buffer saline (PBS, pH 7.4) degassed by purging with nitrogen gas. The solutions were used immediately after preparation to prevent uncontrolled oxidation.

*OEF and LOEF as Substrate for Stem Cell Culturing and Differentiation*: The hNSCs used in this study were human ventral mesencephalic cells (hVM1). Both naïve and optogenetic hNSCs were cultured identically as described before.^12^, ^38^ The hNSCs were cultured for at least two passages after thawing before being seeded on Geltrex (ThermoFisher Scientific) coated OEF/LOEF. Cells were seeded in the same medium that was used before for maintenance, named as growth medium. Growth medium contained DMEM/F12 (ThermoFisher Scientific) with GlutaMAX (ThermoFisher Scientific) as basis. Additionally, the medium contained 6 g L^−1^ glucose (Sigma‐Aldrich), 5 × 10^−3^ m HEPES (ThermoFisher Scientific), 0.5% m m^−1^ AlbuMAX (ThermoFisher Scientific), 40 × 10^−6^ m each of l‐alanine (MerckMillipore), l‐asparagine monohydrate (MerckMillipore), l‐aspartic acid (MerckMillipore), l‐glutamin acid (MerckMillipore), and l‐proline (MerckMillipore), 100× diluted N‐2 supplement (ThermoFisher Scientifica), penicillin/streptomycin mix, and 20 ng L^−1^ each of EGF and FGF (R&D systems).

24 h after seeding, growth medium was replaced with differentiation medium. Differentiation medium was prepared using the same components as growth media, although EGF and FGF were substituted by 1 × 10^−3^ m dibutyryladenosine 3′, 5′‐cylic monophosphate sodium salt (Sigma‐Aldrich) and 2 ng L^−1^ GDNF (PeproTech). After 48 h, the differentiation medium was completely replaced with fresh differentiation medium. Then, 2/3 differentiation medium was replaced with fresh differentiation medium every other day. The differentiation was concluded at 10 days in vitro after seeding and it was the time point for the analysis.

*Detection of Dopamine Exocytosis*: To detect dopamine exocytosis using chronoamperometry, the cell culture medium was replaced with a baseline buffer (pH 7.4), containing 10 × 10^−3^ m HEPES, 5 × 10^−3^ m glucose, 1.2 × 10^−3^ m magnesium chloride, 2 × 10^−3^ m calcium chloride, 150 × 10^−3^ m sodium chloride, and 5 × 10^−3^ m potassium chloride (pH 7.4). For the optical depolarization, a custom‐made optical stimulation setup was created in house using a blue laser diode (PL450B rated 80 mW, Osram Opto Semiconductors Inc.) as the light source. The laser diode was powered by the Rigol DP711 (Batronix GmbH & Co. KG). Light from the laser diode was coupled into the OEF/LOEF using an FC/PC to ferrule patch cable (M81L01, Thorlabs Inc.). Chemical depolarization of the neurons was done by adding a stimulation buffer into the baseline buffer to elevate the final K^+^ concentration to 150 × 10^−3^ m.

*Viral Transfection of hNSCs*: High‐titer of third generation lentiviral particles containing hSyn1‐ChR‐2(H134R)‐mCherry‐WPRE was generated as previously described with a titer of 1.75 × 10^8^ U mL^−1^.^39^ hNSCs were seeded at 80% confluence in a T75 flask for its posterior infection. One day after seeding, cells were infected O/N with 5 µL of the lentivirus in a final volume of 10 mL of proliferation medium. After this first infection, the medium was removed and cells were reinfected again O/N using the same conditions. The next day, medium was discarded and cells, referred to as hNSC‐ChR‐2 line, were allowed to recover for 24 h and were expanded routinely.

*Immunocytochemistry and Confocal Microscopy*: At the experimental endpoint (differentiation day 10), cells were fixed with 4% paraformaldehyde for 15 min. Following thorough rinsing with PBS, cells were incubated with 5% goat serum, 5% horse serum, and 0.25% Triton X‐100 in PBS in order to permeabilize cell membranes and prevent unspecific binding of antibodies. Cells were then incubated with antityrosine hydroxylase (rabbit, PelFreez Biologicals P40101, 1:1000) and anti‐β‐tubulin (mouse, Sigma‐Aldrich T8660, 1:1000) primary antibodies overnight at 4 °C. Following rinsing in PBS, cells were incubated with Alexa 546 goat antimouse (ThermoFisher A‐11030, 1:500) and Alexa 647 goat antirabbit (ThermoFisher A‐21245, 1:500) secondary antibodies for 2 h at room temperature. The cell nuclei were counterstained with 2 µg mL^−1^ Hoechst 33342 (ThermoFisher H1399) for 15 min followed by thorough rinsing with PBS. Confocal laser scanning microscopy was performed on an LSM 710 (Carl Zeiss, Jena, Germany) inverted microscope equipped with a 10× objective. Z‐stacks were acquired from the top to the middle of the fiber in order to visualize the cells growing at different focal planes. Images were processed using ImageJ (NIH) software.

## Conflict of Interest

The authors declare no conflict of interest.

## Supporting information

Supporting Information
