# Supplementary material for: Leaky Optoelectrical Fiber for Optogenetic Stimulation and Electrochemical Detection of Dopamine Exocytosis from Human Dopaminergic Neurons
Source: Adv Sci (Weinh). 2019 Oct 16;6(24):1902011. doi: 10.1002/advs.201902011 (PMC6918109; doi:10.1002/advs.201902011)
Supplement: Supplementary file 1 — Supporting Information [file ADVS-6-1902011-s001.pdf]

Copyright WILEY-VCH Verlag GmbH & Co. KGaA, 69469 Weinheim, Germany, 2019.

ADVANCED SCIENCE
Open Access

Supporting Information

for *Adv. Sci.,* DOI: 10.1002/advs.201902011

Leaky Optoelectrical Fiber for Optogenetic Stimulation and Electrochemical Detection of Dopamine Exocytosis from Human Dopaminergic Neurons

*Shashank Vasudevan, Janko Kajtez, Ada-Ioana Bunea, Ana Gonzalez-Ramos, Tania Ramos-Moreno, Arto Heiskanen, Merab Kokaia, Niels B. Larsen, Alberto Martínez-Serrano, Stephan S. Keller, and Jenny Emnéus**

DOI: 10.1002/ ((please add manuscript number))

Supplementary Information

Leaky Opto-Electrical Fiber for Optogenetic Stimulation and Electrochemical Detection of Dopamine Exocytosis from Human Dopaminergic Neurons

*Shashank Vasudevan, Janko Kajtez, Dr. Ada-Ioana Bunea, Ana Gonzalez-Ramos, Dr. Tania R. Moreno, Dr. Arto Heiskanen, Prof. Merab Kokaia, Prof. Niels B. Larsen, Prof. Alberto Martínez-Serrano, Dr. Stephan S. Keller and Prof. Jenny Emnéus**

S. Vasudevan, J. Kajtez, Dr. A. Heiskanen, Prof. J. Emnéus

Department of Biotechnology and Biomedicine (DTU Bioengineering)

Produktionstorvet, Building 423, Room 122, 2800 Kgs. Lyngby, Denmark

E-mail: jemn@dtu.dk

Dr. A.I Bunea, Dr. S.S. Keller

National Centre for Nano Fabrication and Characterization (DTU Nanolab)

Ørsteds Plads, Building 347, 2800 Kgs. Lyngby, Denmark

Prof. N.B. Larsen

Department of Health Technology (DTU Health Tech)

Produktionstorvet, Building 423, 2800 Kgs. Lyngby, Denmark

A. Gonzalez-Ramos, Prof. M. Kokaia

Epilepsy Centre, Department of Clinical Sciences,

Lund University Hospital, 221 84 Lund, Sweden

Dr. Tania Ramos Moreno

Lund Stem Cell Center, Division of Neurosurgery, Department of Clinical Sciences

Lund University, 221 84 Lund, Sweden.

Prof. A. Martinez-Serrano

Department of Molecular Biology, Universidad Autónoma de Madrid, and Department of Molecular Neuropathology, Center of Molecular Biology Severo Ochoa (UAM-CSIC).

Nicolás Cabrera 1, 28049-Madrid. Spain

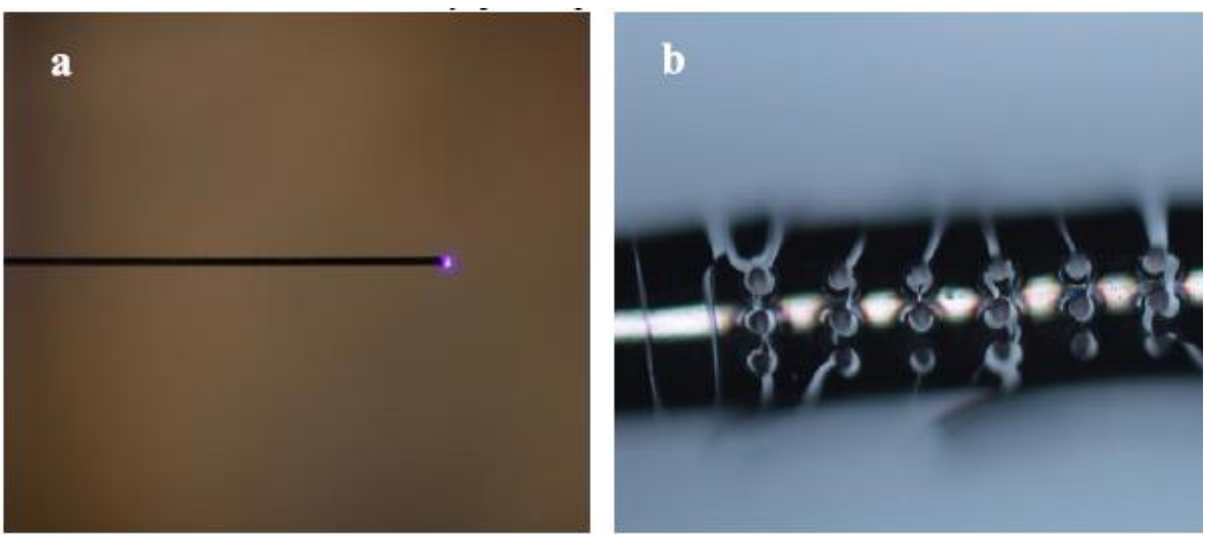

Figure S1: (a) Image showing uniform coverage of pyrolytic carbon on an OEF. **(b)** Cracking in the pyrolytic carbon layer when laser ablation is performed before pyrolysis.

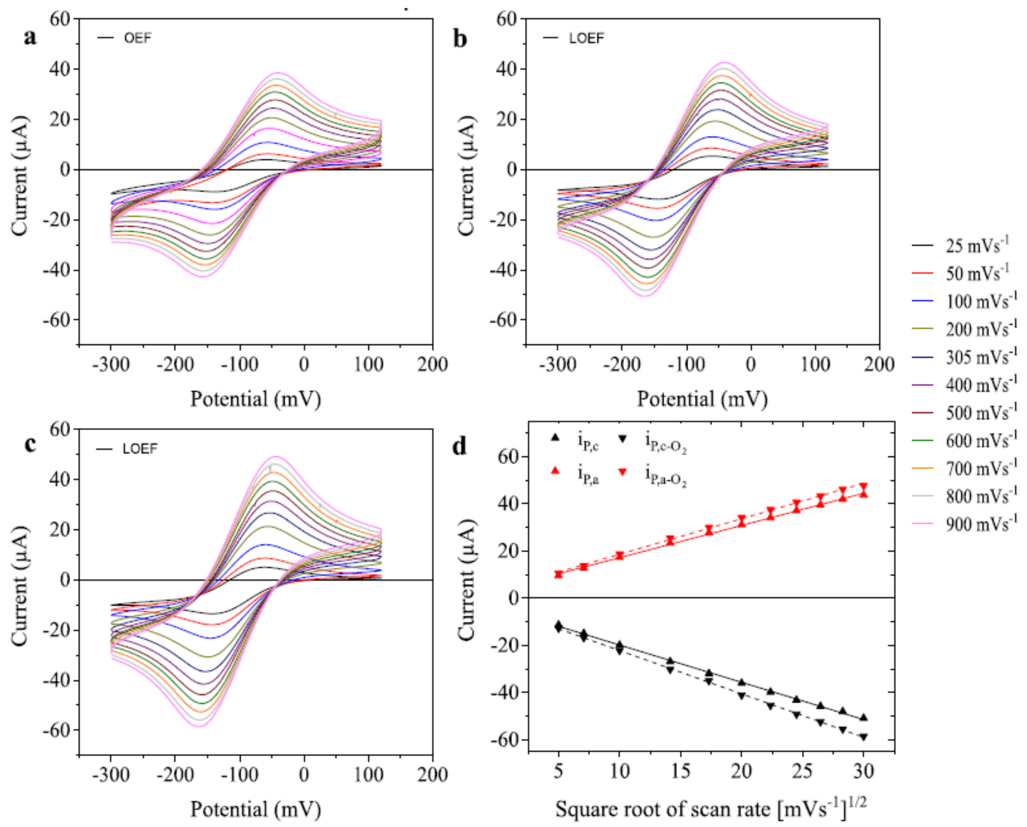

Figure S2. Characteristic cyclic voltammograms of 1mм $[Ru(NH_3)_6]^{2+}$ at multiple scan rates. **(a)** OEF and **(b)** LOEF before O_2 plasma treatment. **(c)** LOEF after O_2 plasma treatment. **(d)** i_p vs. square root of scan rate for LOEF before and after O_2 plasma treatment.

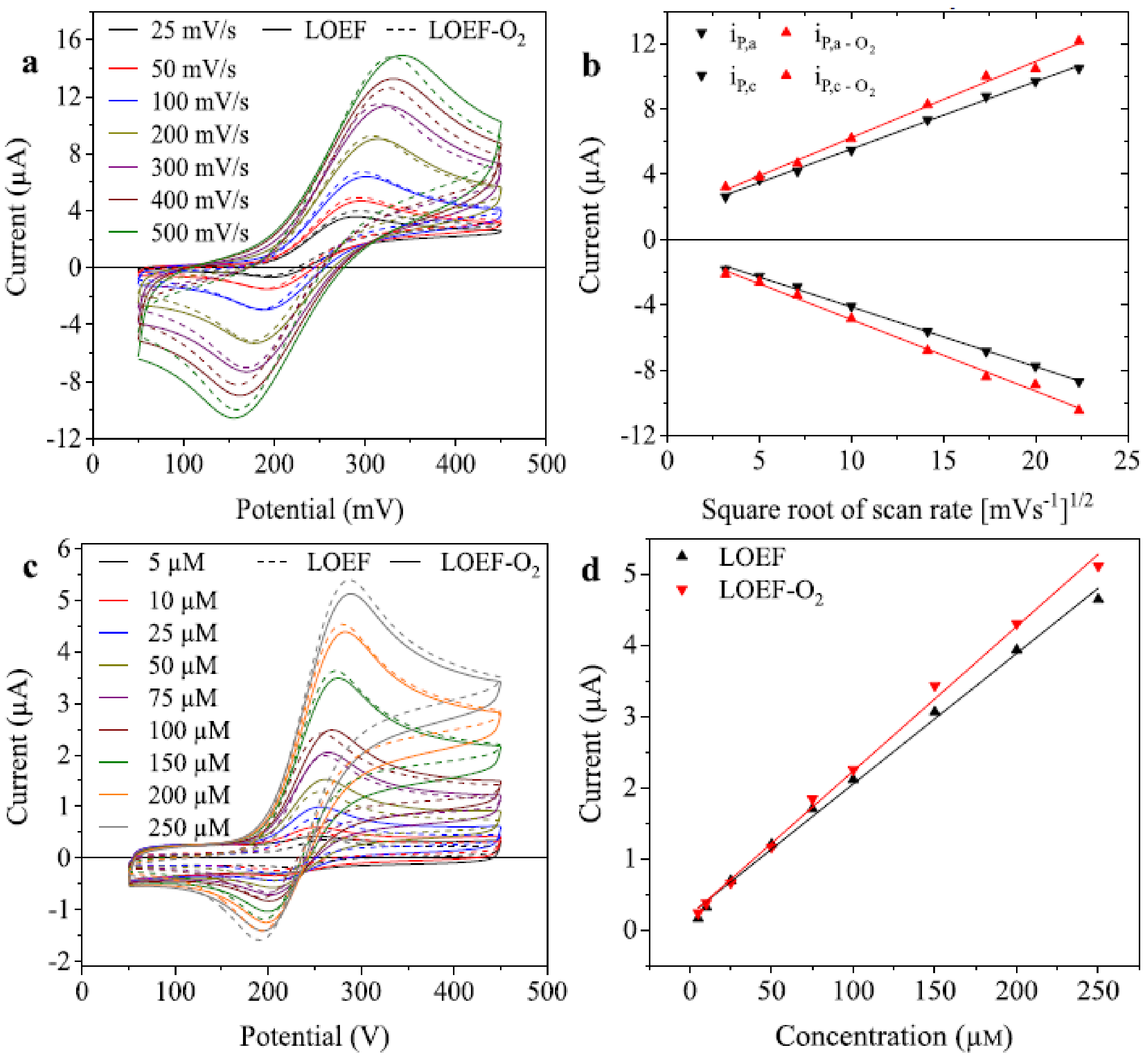

Figure S3. Characteristic cyclic voltammograms of dopamine on a LOEF. **(a)** Multiple scan rates and **(b)** i_p vs. square root of scan rate with 250 μM dopamine before and after O_2 plasma treatment. **(c)** Different dopamine concentrations and **(d)** $i_{P,a}$ vs. concentration acquired at 50 mVs^{-1}.

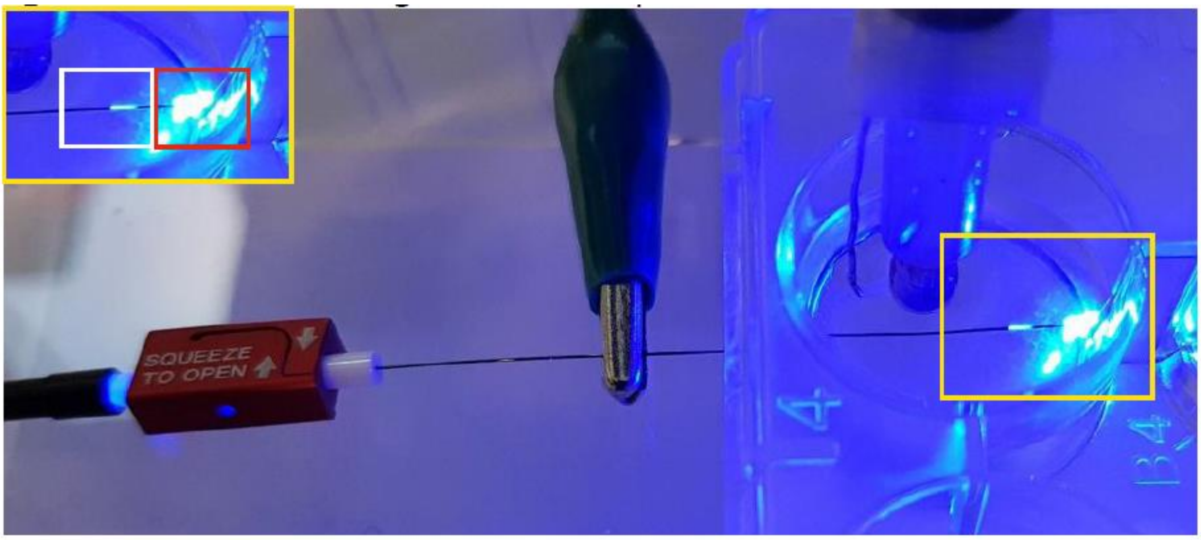

Figure S4. Setup for optogenetic experiments. **(Inset)** Shows leaky region (white box) and light from the tip (red box) of the LOEF under test.
